# Balanced Turbo Field Echo with Extended *k*-space Sampling: A Fast Technique for the Thoracic Ductography

**DOI:** 10.2463/mrms.tn.2015-0111

**Published:** 2016-03-21

**Authors:** Takakiyo NOMURA, Tetsu NIWA, Toshiki KAZAMA, Tatsuya SEKIGUCHI, Takashi OKAZAKI, Shuhei SHIBUKAWA, Hiroaki NISHIO, Makoto OBARA, Yutaka IMAI

**Affiliations:** 1 Department of Radiology, Tokai University School of Medicine, 143 Shimokasuya, Isehara, Kanagawa 259-1193, Japan; 2 Department of Radiology, Tokai University Hospital; 3 Department of Radiology, Gifu Prefectural Tajimi Hospital; 4 Philips Electronics Japan Ltd.

**Keywords:** thoracic duct, magnetic resonance imaging, 3D balanced turbo field echo, real reconstruction

## Abstract

We evaluated the visibility of the thoracic duct by fast balanced turbo field echo with extended *k*-space sampling (bTFEe). The thoracic duct of 10 healthy volunteers was scanned by bTFEe using a 1.5-T magnetic resonance imaging (MRI), which was acquired in approximately 2 minutes. Three-dimensional (3D) turbo spin-echo (TSE) was obtained for comparison. The thoracic duct including draining location of the venous system was overall well visualized on bTFEe, compared to TSE.

## Introduction

The thoracic duct is a central lymphatic system located in the posterior mediastinum in the lower part of the chest connecting to the subclavian region. It contributes to transport excess liquid and protein from the interstitial tissue, and lipid and lipid-soluble vitamins from the gastrointestinal tract, to the circulatory system.^1^ The anatomic variation of the thoracic duct is common, including divided channel, duplication, bilateral outflow, and right outflow.^1–3^ Therefore, it is important to know the course of the thoracic duct especially before the thoracic surgery. The damage of the thoracic duct may cause chylothorax, leading to serious metabolic, immunologic, and nutritional complications. These complications result in a relatively high mortality rate of up to 50% unless treated properly.^4^

The imaging of the thoracic ductography is challenging. Conventional lymphography with the contrast material or lymphoscintigraphy has been performed to visualize the thoracic duct. However, these techniques have some drawbacks, including an invasive method, a relatively long examination time, patients’ discomfort, and low tissue resolution. Computed tomography (CT) is not also useful for visualizing the thoracic duct because of a low tissue contrast of the thoracic duct to the surrounding soft tissue structures,^5^ though CT provides high spatial resolution images.

Recently, the visualization of the thoracic duct by the magnetic resonance imaging (MRI) has been attempted.^1–3,6,7^ An MRI is a noninvasive method and offers morphological details of the thoracic structures. Magnetic resonance thoracic ductography (MRTD) has been performed with single shot MRI sequence because of the many moving organs in the thoracic region. Previous investigators performed the MRTD mainly with heavily T_2_-weighted turbo spin-echo (TSE) sequence.^1–3,6,7^ They showed a relatively good visualization of the thoracic duct with TSE. However, MRTD with TSE has some disadvantages. One of them is a low tissue contrast of the surrounding structures such as the esophagus, the aorta, and the azygous vein. Another disadvantage of MRTD with TSE may be a relatively long scanning time. Since this application requires multiple slice acquisitions to cover targeted anatomy, respiratory gating is necessary for data acquisition; breath holding is impossible for acquiring required number of slices. Therefore, scan time becomes long even using single-shot acquisition. In single-shot imaging, obtained data of MR signals is limited to *kx* and *ky* plane. In other words, acquisition is limited to two-dimensional (2D) imaging. In this study, we used new approach using fast balanced turbo field echo 3D *k*-space acquisition. Here, we used an extended *k*-space sampling scheme, acquiring data not only *kx* and *ky*, but also acquiring neighboring *kz* points simultaneously within a shot. Since this acquisition is not limited to 2D *k*-space plane, larger number of sampling points can be obtained within a shot, compared with conventional single-shot sampling scheme, leading to shorter scan time. The purpose of this study was, therefore, to evaluate visibility of the thoracic duct with a new 3D balanced turbo field echo with extended *k*-space sampling (bTFEe).

## Materials and Methods

### Study participants

Ten healthy volunteers (8 men and 2 women; age range, 25 to 44 years) were enrolled in this study. The ethics committee in our institute approved this study. Written informed consent was obtained from each participant. Body mass index (BMI) of the subjects was 18.2–29.7 (mean, 22.6; standard deviation, 3.24).

### Magnetic resonance imaging

An MRTD was performed with a 1.5-T clinical scanner (Achieva Nova Dual; Philips, Best, The Netherlands) with 32-channel-torso-cardiac coils to obtain a high signal-to-noise ratio and a uniform coil sensitivity. The MRI scan was done with supine position, without elevation of the volunteers’ arms. First, sagittal and axial single-shot T_2_-weighted imaging was performed to grasp roughly the location of the thoracic duct. The scanning area of MRTD was determined from the caudal top of the thoracic duct to the level of the diaphragm. Consequently, the scanning area consisted of an oblique coronal plane, as the cranial portion anteriorly and the caudal portion posteriorly. The MRTD was scanned with bellows-based respiratory gating. Cardiac gate was not used because of longer acquisition time. To reduce the burden on volunteers, the preparations such as dietary restriction or compression of the supraclavicular fossa were not performed before the MR examination in this study.

MRTD with bTFEe was obtained with a segmented 3D *k*-space acquisition. The actual parameters for bTFEe were as follows: repetition time (TR)/echo time (TE), 4.8/2.4 ms; flip angle, 120 degrees; matrix, 208 × 208; slice thickness, 1.6 mm; turbo factor (number of data sampling per shot), 200; field of view, 304 mm; SENSE factor, 2; the number of slices, 72; number of acquisitions, 1. Centric *k*-space ordering in *ky* direction was set in the acquisition. In conventional scheme, data are acquired in several *ky* points from center to outer space with fixing *kz* line in the shot and continue this acquisition in successive shots by shifting *kz* line sequentially. In bTFEe, sampling points within a shot is extended from the *ky* points on the single *kz* line to neighboring *kz* points according to the number of turbo factors. It means number of turbo factors can be set larger than the number of *ky* points in single *kz* line, leading to shorter scan time compared to that of conventional single-shot acquisition scheme. The number of data sampling for *ky* and *kz* per shot was automatically determined according to the turbo factor; approximately three *kz* lines were obtained per shot at the current turbo factor setting. Four dummy pulses were used in the α/2 approach to achieve steady-state conditions. Data for total 3D *k*-space on bTFEe was segmentally sampled with respiratory gating. Shot interval was determined according to the respiratory gate on each subject. Volume shimming was used by applying a volume of interest to the upper mediastinum, avoiding the lung region, to improve magnetic field homogeneity. In place of fat suppression (i.e., spectral presaturation with inversion recovery or spectral attenuation with inversion recovery), real reconstruction, which displays the positive and negative signal values,^8^ was performed to suppress the signal of the fat tissue. Data were reconstructed in a final resolution of 0.6 × 0.6 × 0.8 mm.

An MRTD using single-shot 3D heavily T_2_-weighted TSE was also performed for comparison, in the same scanning area at bTFEe. The parameters of MRTD with 3D TSE were as follows: TR/TE, 4000/600 ms; flip angle, 90 degrees; matrix, 288 ×288; slice thickness, 1.6 mm; turbo factor, 89; field of view, 350 mm; SENSE factor, 2; the number of slices, 72; half scan factor, 0.6; spectral presaturation with inversion recovery; number of acquisitions, 1. Data were reconstructed in a final resolution of 0.68 × 0.68 × 0.8 mm.

### Image analysis

The thoracic duct has been known as a water-intensity structure extending in the craniocaudal direction between the descending thoracic aorta and the azygos vein at the level of the lower thoracic region, connecting to the subclavian region.^1–3,5–7^ The visibility of the thoracic duct on each MR sequence was assessed by two board-certified radiologists with experience of the thoracic imaging more than 10 years with consensus achieved by discussion. Before image analysis, the thoracic duct from the cranial top to the level of the diaphragm was trisected: the upper, middle, and lower sections. The visibility of the thoracic duct of the each section on bTFEe and TSE was assessed by using a five-point scale: 0 = no visualization; 1 = partial visualization (partial visualized thoracic duct, but most of the thoracic duct is not visible); 2 = moderately visualization (about half of the thoracic duct is visualized); 3 = good visualization (most of the thoracic duct is visualized, but partially impossible to identify the continuity of the thoracic duct); and 4 = completely visualized (no discontinuity of the thoracic duct). The assessment of the images was done by seeing all the source oblique coronal images on a PACS viewer (SDS viewer; Techmatrix, Tokyo). These assessments were done for bTFEe and TSE, respectively, in a random order. The scores of each section on each sequence were compared between bTFEe and TSE.

Since bTFEe is based on balanced field echo, bTFEe visualizes not only the thoracic duct but also the blood vessels. Therefore, the draining location of the thoracic duct to the venous system in the left subclavian region on bTFEe was also assessed using a three-point scale: 0 = impossible to identify the draining location; 1 = probable identification of the draining location; 2 = possible to identify the draining location.

The scores for visibility of the thoracic duct on bTFEe and TSE were compared using Wilcoxon test. *P* values < 0.05 were considered to indicate a statistically significant difference. Statistical analyses were performed with a software package (MedCalc, version 15.8; MedCalc Software, Mariakerke, Belgium).

Source images were transferred to a workstation (Ziostation; Ziosoftware, Tokyo). A board-certified radiologist with experience of the thoracic imaging more than 10 years created representative images with a curved planar reformation and a partial maximum intensity projection.

## Results

The acquisition time for bTFEe ranged from 1 minute 31 seconds to 2 minutes 19 seconds; that for TSE ranged from 6 minutes 30 seconds to 8 minutes 1 second.

**Fig. 1.**
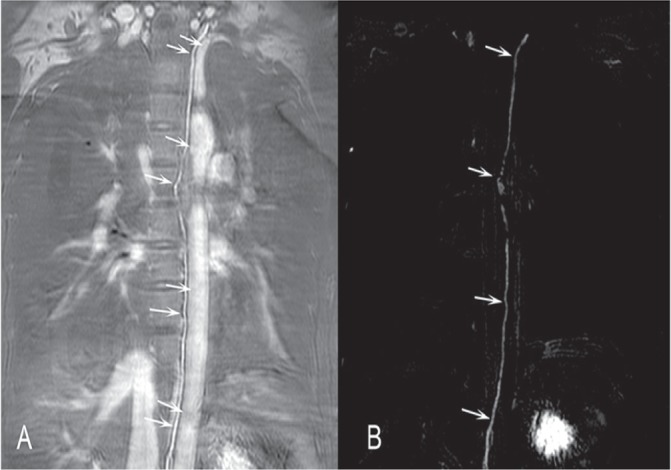
Magnetic resonance thoracic ductography in a 26-year-old woman. The thoracic duct (arrows) is well visualized on both balanced turbo field echo with extended *k*-space sampling (bTFEe) (**A**) and three-dimensional turbo spin-echo (**B**), with a curved planar reformation. Note that bTFEe shows not only the thoracic duct, but the surrounding structures such as the vessels and the vertebras.

**Fig. 2.**
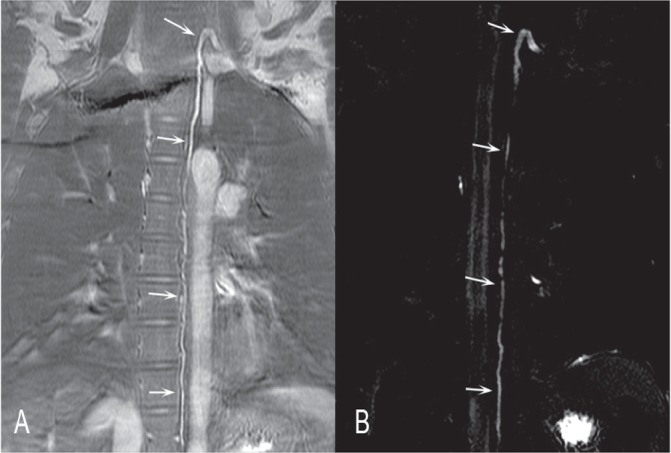
Magnetic resonance thoracic ductography in a 29-year-old man. Balanced turbo field echo with extended *k*-space sampling with a curved planar reformation (**A**) well visualizes the thoracic duct in all the segments (arrows). Three-dimensional turbo spin-echo with a curved planar reformation (**B**) less visualizes the thoracic duct (arrows) in the upper section near the aortic arch.

Table 1 summarizes the scores of visualization of the thoracic duct on bTFEe and TSE. Both sequences visualized the thoracic duct from moderately to completely (Fig. 1). The scores of bTFEe achieved 3 or 4 in all the sections of the thoracic duct, except for a case in the lower section showing a score of 2. The scores of TSE achieved from 1 to 4. The middle and lower section of the thoracic duct was relatively well visualized on TSE (i.e., median scores of 3, 4, respectively), but the upper section of the thoracic duct was less visualized (median score of 2). The scores of bTFEe were significantly higher than those of TSE in the upper and middle sections (*P* = 0.004, 0.008, respectively) (Fig. 2). The visibility of the lower section of the thoracic duct was not significantly different between bTFEe and TSE.

**Fig. 3.**
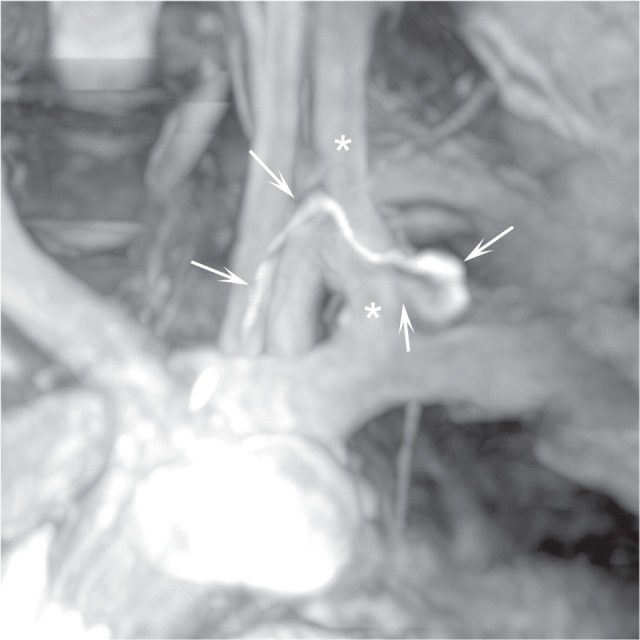
The visualization of the draining location of the thoracic duct in the subclavian region in a 26-year-old woman. Balanced turbo field echo with extended *k*-space sampling with a partial maximum intensity projection well visualizes the thoracic duct (arrows) draining to the proximal portion of the internal jugular vein (*).

The draining location of the thoracic duct to the venous system in the subclavian region on bTFEe was almost well identified (Fig. 3); most of the volunteers achieved a score of 2, and only one volunteer’s score was 1.

## Discussion

We found a relatively good demonstration of the thoracic duct using bTFEe. For this reason, we assume that the rapid 3D *k*-space sampling on bTFEe in segmented slabs may contribute to both a reduction of the motion artifact and the continuous visibility of the thoracic duct. In conventional balanced turbo field echo sequence, maximum number of sampling points within a shot (or single respiratory cycle) is limited to number of *ky* points in single *kz* line. Whereas, in bTFEe, larger points are acquired, including several *kz* points on each respiratory gating, resulting in faster scanning method Although 3D simultaneous *k*-space acquisition can be a rapid signal acquisition method, the continuous acquisition of the entire slab to cover the entire course of the thoracic duct needs more than one minute, which should result in containing motion artifact. We set segment acquisition in the slab, and obtained the data under respiratory gate. The balance of the combination between segmentation of the slab area and the acquisition time may be important for the good visualization of the thoracic duct on bTFEe.

To date, MRTD has been performed mainly with heavily T_2_-weighted TSE. The advantage to use TSE is that the course of the entire thoracic duct is easily understood with a maximum-intensity projection because other structures without high water content are suppressed. Whereas, there are some disadvantages of TSE technique for MRTD. First, the relation between the thoracic duct and the surrounding structures are difficult to be identified. Second, TSE with respiratory gate needs a relatively long acquisition time.^1–3,7^ Third, half Fourier imaging on TSE results in blurring of the structures. On the other hand, bTFEe visualizes not only the thoracic duct, but organs such as the vessels and vertebra, enabling to grasp the anatomical relationship between the thoracic duct and the surrounding structures. Therefore, bTFEe may be more effective than TSE in the situation such as a preoperative evaluation of the esophagus, the vertebra, and the vessels near the thoracic duct. Furthermore, the acquisition time of bTFEe is faster than that of TSE. This means that bTFEe is more effective in use to the patients with severe condition in the clinical practice than TSE.

The utilities of balanced field echo for MRTD has not been well investigated. We found only one literature describing the utilities of 3D balanced turbo field echo with navigator gate and cardiac-trigger by Kato et al.^5^ They reported generally good visualization of the thoracic duct. However the upper portion of the thoracic duct was less visualized with 3D balanced turbo field echo. They suggested that balanced turbo field echo may be susceptible for artifact at the air-tissue or bone-tissue boundaries. Meanwhile, bTFEe showed a relatively good visualization of the upper portion of the thoracic duct. Although the techniques including bTFEe, shimming method, and real reconstruction might have contributed to improve the visibility of the thoracic duct, we did not compare the visibility of the thoracic duct between conventional balanced turbo field echo and bTFEe in this study; the difference in visibility of the thoracic duct between both methods remains unclear. However, bTFEe with respiratory gate has an advantage of shorter acquisition time than balanced turbo field with respiratory and cardiac gate echo.

Since movements of the heart, vessels, and thorax severely damage the imaging quality, respiratory or cardiac gate is mandatory for MRTD. Previous studies^2,3,7^ mainly used only respiratory gating. However, other methods included electrocardiographic gating with intermittent breath-holding,^6^ and simultaneous use of both respiratory and cardiac gating.^5^ Takahashi et al.^1^ investigated several gating methods including intermitted breath holding after expiration or inspiration, and respiratory gating. They concluded that respiratory gating was better than intermitted breath holding. In this study, we used only respiratory gate for bTFEe. Although we did not attempt the other possible gating method for bTFEe, we believe respiratory gating is appropriate method with bTFEe for MRTD. In addition, examinees may feel more comfortable with the use of respiratory gating than intermittent breath holding.

In this study, bTFEe showed good visualization of the thoracic duct in the subclavian region connecting to the venous system. Since complex variations of the draining portion of the thoracic duct to the veins are known,^2,3^ bTFEe is useful to understand the detail structure of the thoracic duct draining to the venous system. Recently percutaneous transvenous embolization of the thoracic duct through the subclavian vein was reported for patients with malformation of the thoracic duct and bleeding tendency.^9^ Therefore, bTFEe can be a useful technique to assess this connection before percutaneous transvenous embolization of the thoracic duct.

Previous investigators^3^ used supraclavicular compression to obtain the better visualization of the thoracic duct. Although we did not use such materials, the thoracic duct was well visualized on bTFEe. As far as healthy subjects are scanned, we assume that no supraclavicular compression is needed for MRTD on bTFEe. Furthermore, no supraclavicular compression might have contributed to the better visualization of the draining portions of the thoracic duct in the venous system in this study. On the other hand, supraclavicular compression may improve visibility of MRTD, particularly in elderly patients with narrow thoracic duct.

On steady-state free precession (SSFP) imaging, resonant frequency of lipid has opposite phase to that of water, when selecting certain TR (i.e., TR = 4.6 ms at 1.5T).^10^ This phenomenon can be applied to fat-suppression technique that does not increase scan time. As such, fat signal should be negative value, whereas water signal should be positive, on real reconstruction images (i.e., images maintaining the positive and negative signals values) on SSFP, resulting in signal difference between water and fat. Meanwhile, this signal difference should decrease on magnitude reconstruction images, where both water and fat signals are displayed in absolute value. Thus, real reconstruction technique might have some effect on suppressing fat tissue in this study, because fat tissue in the posterior mediastinum around the thoracic duct showed a relatively low intensity on bTFEe. However, we did not sufficiently assess the utility of fat suppression on bTFEe using real reconstruction technique, and this should be evaluated in the future. In addition, the method for *k*-space ordering might have affected the imaging contrast for MRTD. Morita et al.^11^ compared the contrast of the several structures in the upper abdomen between centric and linear *k*-space ordering on 3D SSFP, and reported that the contrast for vessels and fat decreased on centric *k*-space ordering compared to linear *k*-space ordering, while that for the bile duct was not significantly different between these *k*-space ordering methods. Although we assumed that visualization of both the thoracic duct and surrounding structures has an advantage on balanced turbo field echo imaging, prominent visibility of the tissues around the thoracic duct might interfere the correct interpretation of the thoracic duct. Therefore, we set centric *k*-space ordering in this study. However, we used dummy pulses applied in the α/2 approach to achieve steady-state conditions; the selection of *k*-space ordering might have less effect for the imaging contrast in this study.^12^

Only one case showed poor visualization in the lower section of the thoracic duct on bTFEe. In this case, the lower section of thoracic duct was visualized as the low signal structure. This is expected to the reversed signal on the real image due to the magnetic inhomogeneity or inadequate shimming, particularly at the periphery in the scanned area, because the BMI of this case was the highest (i.e., 29.7) among the subjects. Another shimming method such as pencil beam second-order shimming might have improved the visibility of the thoracic duct. In addition, simultaneous observation of both real and magnitude images may be helpful to reduce the misreading of the thoracic duct in such a case because magnitude image show the data in absolute value.

This study has several limitations. First, there were no references of the thoracic duct in this study. We consider the thoracic duct with the knowledge of the anatomical location and previous reports.^2,3^ However, it was indistinguishable whether the partial discontinuity on MRTD was due to the imaging artifact or physiological changes such as contraction of the smooth muscle of the lymphatic wall.^7^ Second, subjects consisted of relatively young healthy volunteers. A further study is necessary to assess the reproducibility of the results in elderly subjects. Third, we did not perform quantitative analysis such as measuring the diameter of the thoracic duct, because TSE may contain blurring. Only a visual inspection was performed in this study.

In conclusion, bTFEe can visualize the entire course of the thoracic duct including the draining location to the venous system clearly in a relatively short acquisition time.

## Figures and Tables

**Table 1.** The assessed score regarding the visibility of each section of the thoracic duct

Section of the thoracic duct	bTFEe	TSE	*P* value
Upper	4 (4)	2 (1–4)	0.004
Middle	4 (3–4)	3 (1–3)	0.008
Lower	4 (2–4)	4 (1–4)	1.00

Values are the median scores, and range in the parenthesis, rated by two radiologists. Scores closer to 4 indicate good visibility, whereas scores closer to 0 indicate poorer visibility of the thoracic duct. bTFEe, balanced turbo field echo with extended *k*-space sampling; TSE, turbo spin-echo.
